# MicroRNA-7 Inhibits Tumor Metastasis and Reverses Epithelial-Mesenchymal Transition through AKT/ERK1/2 Inactivation by Targeting EGFR in Epithelial Ovarian Cancer

**DOI:** 10.1371/journal.pone.0096718

**Published:** 2014-05-09

**Authors:** Xingchen Zhou, Yuan Hu, Lan Dai, Yunfei Wang, Jinhua Zhou, WenWen Wang, Wen Di, Lihua Qiu

**Affiliations:** 1 Department of Gynecology and Obstetrics, Renji Hospital, School of Medicine, Shanghai Jiao Tong University, Shanghai, China; 2 Shanghai Key Laboratory of Gynecologic Oncology, Shanghai, China; Seoul National University, Korea, Republic Of

## Abstract

Epidermal growth factor receptor (EGFR) overexpression and activation result in increased proliferation and migration of solid tumors including ovarian cancer. In recent years, mounting evidence indicates that EGFR is a direct and functional target of miR-7. In this study, we found that miR-7 expression was significantly downregulated in highly metastatic epithelial ovarian cancer (EOC) cell lines and metastatic tissues, whereas the expression of, EGFR correlated positively with metastasis in both EOC patients and cell lines. Overexpression of miR-7 markedly suppressed the capacities of cell invasion and migration and resulted in morphological changes from a mesenchymal phenotype to an epithelial-like phenotype in EOC. In addition, overexpression of miR-7 upregulated CK-18 and β-catenin expression and downregulated Vimentin expression, accompanied with EGFR inhibition and AKT/ERK1/2 inactivation. Similar to miR-7 transfection, silencing of EGFR with this siRNA in EOC cells also upregulated CK-18 and β-catenin expression and downregulated Vimentin expression, and decreased phosphorylation of both Akt and ERK1/2, confirming that EGFR is a target of miR-7 in reversing EMT. The pharmacological inhibition of PI3K-AKT and ERK1/2 both significantly enhanced CK-18 and β-catenin expression and suppressed vimentin expression, indicating that AKT and ERK1/2 pathways are required for miR-7 mediating EMT. Finally, the expression of miR-7 and EGFR in primary EOC with matched metastasis tissues was explored. It was showed that miR-7 is inversely correlated with EGFR. Taken together, our results suggested that miR-7 inhibited tumor metastasis and reversed EMT through AKT and ERK1/2 pathway inactivation by reducing EGFR expression in EOC cell lines. Thus, miR-7 might be a potential prognostic marker and therapeutic target for ovarian cancer metastasis intervention.

## Introduction

Ovarian cancer is the major cause of deaths from gynecologic malignancies and the 5th leading cause of cancer-related deaths among women in the world [1]. According to the national cancer institute (NCI) report, about 22280 new cases will be diagnosed with ovarian cancer in America in 2012, and 15500 patients will die of this disease, and the 5-year survival rate for them is about 30%. It has been speculated that metastasis remains the leading cause of relapse and death from ovarian cancer, and yet the molecular mechanisms associated with acquisition of metastatic ability in human ovarian cancer are poorly understood.

MicroRNAs (miRNAs) are a class of small non-coding RNA of approximately 20–22 nucleotides long that function as post-transcriptional regulators by targeting 3′ untranslated regions (UTR) of mRNAs and causing either inhibition of translation or degradation of mRNA [2]. MiRNAs contribute to diverse cellular processes including proliferation, apoptosis, invasion and morphogenesis [3], [4], [5]. Moreover, a range of miRNAs have been identified that function as classical oncogenes or tumor suppressor genes [6], [7], [8]. MiR-7 has been characterized as a tumor suppressor in several human cancers. It targets a number of proto-oncogenes, including insulin-like growth factor-1 receptor (IGF1R) [9] epidermal growth factor receptor (EGFR) [10], p21-activated kinase 1 (Pak1) and associated cdc42 kinase 1 (Ack1) [11]. It′s demonstrated that overexpression of miR-7 inhibited schwannoma cell growth both in culture and in xenograft tumor models in vivo, which correlated with downregulation of EGFR, Pak1 and Ack1 [11].

Approximately 70% of epithelial ovarian cancer (EOC) express activated EGFR [12]. EGFR overexpression and activation result in increased proliferation and migration of solid tumors including ovarian cancer [13]. Activation of EGFR tyrosine kinase results in activation of a number of intracellular signals, which culminate in not only cell proliferation but also other processes that are crucial to cancer progression, including cell migration, angiogenesis, metastasis, and epithelial-mesenchymal transition (EMT). These events are mediated through various downstream targets of EGFR (e.g. protein kinase (AKT) and extracellular signal-regulated kinase 1/2 (ERK1/2)) [14], [15], [16]. Interestingly, it′s shown that miR-7 directly targets EGFR mRNA 3′- UTR, and then inhibits expression of its mRNA and protein [17]. Although EGFR signaling is important and well studied with respect to EOC progression, little is known about how miR-7 mediate EGFR signaling to modulate EOC cell metastasis.

In the present study, we identify for the first time that miR-7 plays an important role in EOC metastasis. Furthermore, we show that miR-7 reverses EMT through AKT/ERK1/2 inactivation by targeting EGFR in EOC, which provides a novel insight into the mechanisms underlying metastasis of ovarian cancer.

## Materials and Methods

### Patients and Ethics

Paired samples of primary epithelial ovarian cancer tissues and metastatic tissues (omentum or peritoneum) were obtained from patients with FIGO stage III-IV advanced EOC who had undergone tumor debulking at Renji Hospital, School of Medicine, Shanghai Jiao Tong University between 2010 and 2012. Among them, 17 paired samples were snap-frozen in liquid nitrogen and stored at −80°C for later RNA extraction, 25 paired samples were Formalin fixed and paraffin embedded. Samples were clinically and pathologically shown to be correctly labeled. The study was approved by the institutional review board of Renji Hospital, School of Medicine, Shanghai Jiao Tong University and written informed consent was obtained from all patients. All clinical investigation was conducted according to the principles expressed in the Declaration of Helsinki.

### Materials

MiR-7 plasmid and negative control (NC) were synthesized by Shanghai IBS Company. The sequence of plasmid and NC are as follows: 5′-UGGAAGACUAGUGAUUUUGUUGU-3′; Negative Control: 5′-GAAATCTACTGCGCGTGGAGAC-3′ (IBS company). TaqMan kit (Applied Biosystems) specified for quantification of miRNA was used to assess the expression of miR-7 and U6. EGF receptor Rabbit mAb (# 4267), Phospho-EGF Receptor (Tyr992) Rabbit mAb (#2235), Phospho-p44/42 MAPK (Thr202/Tyr204) Rabbit mAb (#4370), Phospho-Akt (Ser473/Thr308), Rabbit mAb (#4060/#2965), Akt (pan) Rabbit mAb (#4691), LY294002 (PI3K Kinase Inhibitor) (#9901), U0126 (MEK1/2 Inhibitor) (#9903), Vimentin Rabbit mAb (# 5741), and β-catenin rabbit mAb and E-cadherin Rabbit mAb (# 3195) were purchased from Cell Signaling Technology (CST). cytokeratin-18 (CK-18) mAb (T410) pAb (BS1204) was purchased from Bioworld Technology. P44/42 MAPK Rabbit mAb was obtained from Santa Cruz ((sc-33746)). GAPDH mouse mAb was purchased from ABmart (#M20006). 800CW conjugated goat anti-Rabbit IgG((926-32210), highly cross adsorbed and 800CW conjugated goat anti-mouse IgG(926-32211), highly cross adsorbed were purchased from Li-Cor Biosciences. EGFR and control short interfering RNAs (siRNA) were from Sanong Biotech.Has-miR-7-LNA detection probe were purchased from Exiqon (38485-15).

### Cell culture

HO-8910pm, HO-8910 cell lines were obtained from the Cell Bank of the Chinese Academy of Sciences (Shanghai, China), CAOV-3, SKOV3, A2780, A2780/DDP, and ES-2 cells were obtained from the ATCC (Manassas, VA). Cells were cultured in Roswell Park Memorial Institute 1640 medium (RPMI-1640) medium (Hyclone) supplemented with 10% fetal bovine serum (Hyclone) and Penicillin/Streptomycin (1∶100, Sigma) in a humid atmosphere incubator with 5% CO2 at 37°C. Unless otherwise indicated, cells were grown to 70–80% confluence, then serum-starved overnight in serum-free RPMI-1640 medium prior to treatment.

### miRNA and siRNA Transfection

80–90% confluent cells were transfected with human miR-7 plasmid (miR-7) or negative control (NC) and 30–40% confluent cells were transfected with EGFR siRNA or siRNA-NC by Lipofectamine 2000 (Invitrogen) according to the manufacturer's protocol. Total RNA was extracted 24 hours after transfection, and total cell protein were extracted 48 or 72 hours after transfection.

### Reverse transcription quantitative real-time PCR

Total RNA was isolated by TRIzol Reagent (Invitrogen). Mature miR-7 was reverse-transcribed with specific RT primers, quantified with a TaqMan probe, and normalized by U6 small nuclear RNA using TaqMan miRNA assays (Applied Biosystems). mRNA expression analysis was conducted by quantitative PCR using SYBR green dye, with relative changes calculated by the ΔΔCt method. Primers used were as follows: GAPDH-F, 5-TGCACCACCAACTGCTTAGC-3; GAPDH-R, 5-GGCATGGACTGTGGTCATGAG-3; EGFR-F, 5-AGCCATGCCCGCATTAGCTC-3; EGFR-R, 5-AAAGGAATGCAACTTCCCAA-3.

### Western blotting

Whole cell extracts were prepared as described previously [18] and equal amounts of protein were separated by a 8% or 10% SDS-PAGE and elctrotransferred to a PVDF membrane (Millipore). The membranes were then blocked for 1 hour at room temperature with Li-Cor blocking agent (Li-Cor). With constant shaking, the membranes were incubated with primary antibodies overnight at 4°C followed by incubation for 1 hour with the appropriate secondary antibodies labeled with 800IRdye. Immunoreactivity was detected and quantified with the infrared Odyssey imaging System (Li-Cor).

### Migration assays

Cells (8×10^4^) were harvested and re-suspended in serum-free RPMI-1640 medium and put into the upper wells of the Boyden chamber (Corning). The medium containing 10% fetal bovine serum was added into the lower chamber. After 8h of incubation, cells remaining on the upper surface of the membranes were removed, cells that had invaded through the 8 µm pore size membrane were fixed, stained, and counted under a microscope at 200 × magnification. The results were averaged among three independent experiments.

### Invasion assays

Cells (3×10^4^) were placed into the upper chambers coated with 50 µl of Matrigel (1∶5 dilution in serum-free medium). Medium supplemented with 10% serum was added to the outer cup. After 24 h of incubation, cells remaining on the upper surface of the membranes were removed, cells that had invaded through the Matrigel and the 8 µm pore size membrane were fixed, stained, and counted under a microscope at 200 × magnification. The results were averaged among three independent experiments.

### Immunohistochemistry (IHC) and Chromogenic in situ hybridization(CISH)

Immunohistochemistry (IHC) was performed using the horse-radish peroxidase (HRP)-polymer anti-mouse IHC DAB (diami-nobenzidine)-based kit (MaxVision, Fuzhou, China), according to the manufacturer protocol. Antigen retrieval was performed using borate buffer (pH = 8), followed by incubation in hydrogen peroxide and additional blocking steps. Antibodies were used at 1∶50. The IHC was examined and imaged using an OLYMPUS BX51 microscope (Tokyo, Japan) at 1∶200. Positve signals were identified by the intense brown labeling of their cell membranes.

*Chromogenic in situ hybridization (CISH)* was performed using the Has-miR-7 probe from Exiqon (mercury LNA detection probe 5′ and 3′-DIG (digoxigenin)-labeled). The probe was detected using digoxigenin antibody (Abcam), LSAB2 System-HRP (Dako Denmark A/S, Glostrup, Denmark) and liquid DAB+ Substrate Chromogen System (Dako) according to manufacturer's instruction. The CISH was examined and imaged using an OLYMPUS BX51 microscope (Tokyo, Japan) at 1∶200. Positive hybridization signals were identified by the intense brown labeling of their cell cytoplasms.

The results of IHC and CISH were independently scored by two pathologists in a blind manner. The scoring was based on the intensity and extent of staining and was evaluated according to the following histological scoring method. Staining intensity was graded as follows: 0, negative staining; 1, weak staining; 2, moderate staining; 3, strong staining. The mean proportion of staining cells per specimen was determined semi-quantitatively and scored as follows: 0 for staining <1%, 1 for 1–25%, 2 for 26–50%, 3 for 51–75%, and 4 for >75% of the examined cells. The histological score (H-score) for each specimen was computed by the formula: H-score =  proportion score×intensity score. A total score of 0–12 was calculated and graded as negative (−, score:0), weak (+, score:1–4), moderate (++, score:5–8) or strong (+++,score:9–12).

### Statistical analysis

Statistical analysis was performed with the SPSS 12.0 statistical analysis software package. In each in-vitro experiment, a minimum of three wells/dishes was used and similar results were obtained. Each experiment was repeated a minimum of three times, the mean value of the repetitions was calculated and this value was used in the statistical analysis. Experimental data are expressed as means with standard deviation (SD). The differences between groups were evaluated using Student's t test when comparing only two groups or analyzed by one-way ANOVA with post hoc test when more than two groups were compared. The correlation between EGFR and miR-7 was analyzed using Spearman's rank test. The results of IHC and CISH were analyzed using Wilcoxon test. Differences were considered statistically significant at P<0.05, *P<0.05 and **P<0.01.

## Results

### miR-7 expression is inversely correlated with EOC metastasis

**Figure 1:**
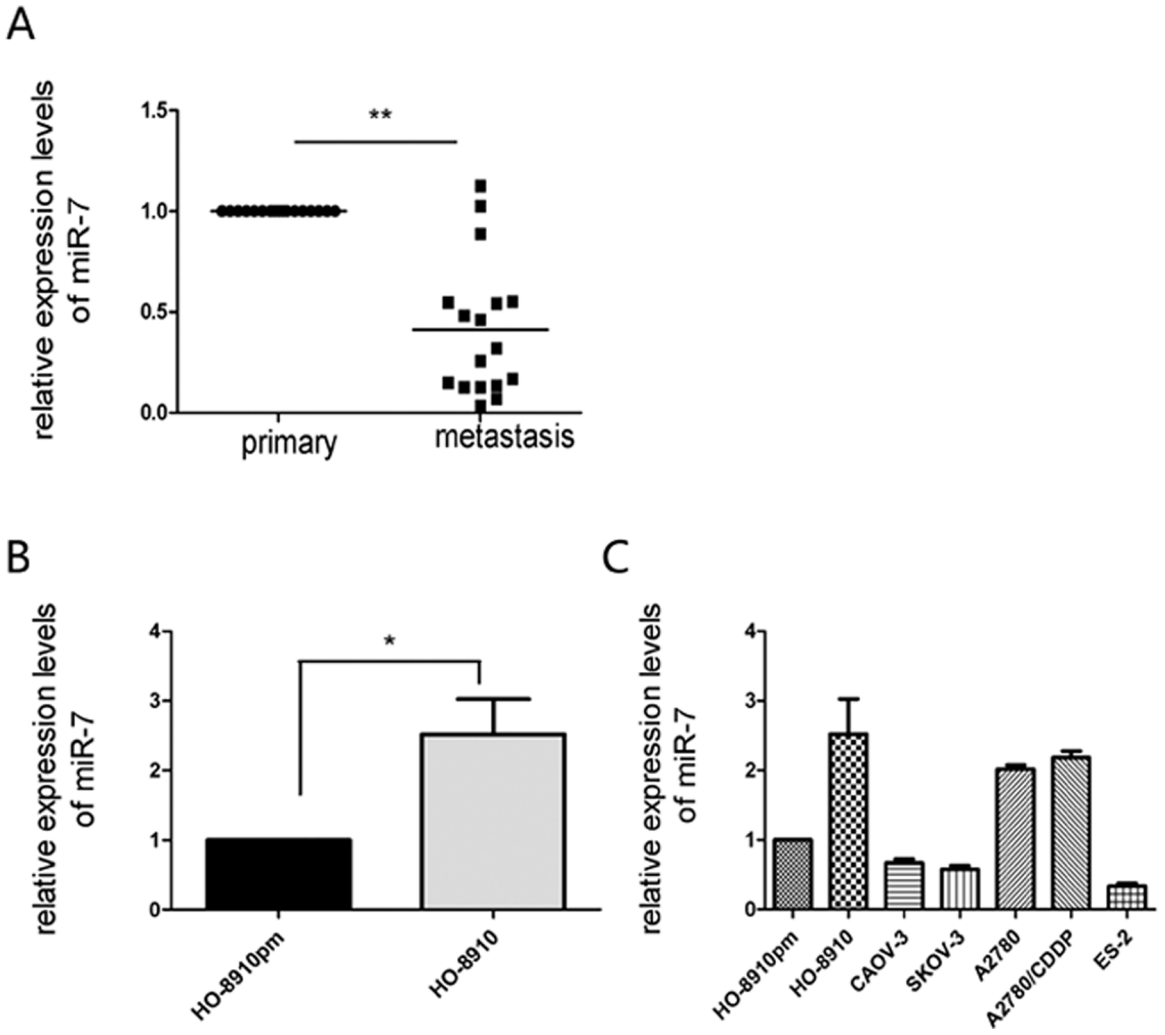
miR-7 expression is inversely correlated with EOC metastasis. (A) The relative expression of miR-7 in 17-paired EOC tissues from omentum or peritoneum metastases and primary EOC tissues was detected by qRT-PCR. The U6 small nuclear RNA was used as an internal control and the fold change was calculated by the ΔΔCt method (B) The expression level of miR-7 in one pair of low and high metastatic EOC cell lines. (C) The expression level of miR-7 in seven EOC cell lines. (*P<0.05. **P<0.01).

To explore the expression and significance of miR-7 in EOC metastasis, we detected miR-7 expression in 17-paired metastatic EOC tissues and primary EOC tissues. Quantitative real-time PCR (qRT-PCR) showed that tissues from omentum or peritoneum metastases expressed lower levels of miR-7 compared with primary EOC tissues, indicating an inverse relationship between the expression of miR-7 and the metastatic status of EOC tissues (Figure 1A). Furthermore, we selected HO-8910 and its highly metastatic clone HO-8910pm. HO-8910pm was established and characterized in Cell Bank, Chinese Academy of Sciences [19], [20], [21]. We found that miR-7 expression was significantly decreased in highly metastatic clone HO-8910pm compared with HO-8910 (Figure 1B). Taken together, our results suggest that down-regulation of miR-7 is correlated with increased EOC metastasis and that miR-7 might suppress EOC progression. To determine the optimal cell lines for further study, we measured the expression of miR-7 in seven EOC cell lines (HO-8910pm, HO-8910, A2780, A2780/DDP, SKOV-3, CAOV-3 and ES-2). Our data showed that the expression of miR-7 was lowest in ES-2 cell line (P<0.05) (Figure 1C), which is another EOC cell with highly metastatic potential. Therefore, we choose HO-8910PM and ES-2 to study the mechanisms of miR-7 inhibiting metastasis in EOC in the following experiments.

### miR-7 downregulates EGFR in EOC cells

**Figure 2:**
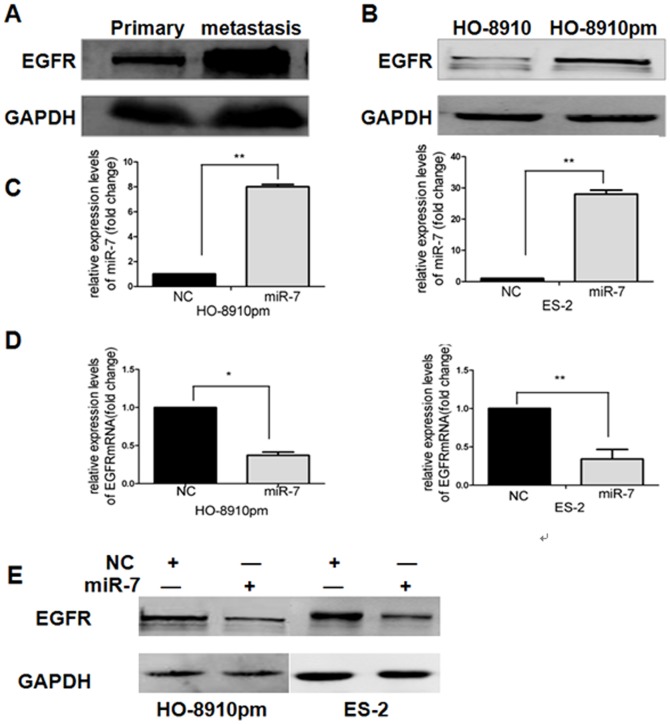
miR-7 downregulates EGFR in EOC cells. (A) The protein expression of EGFR in 17-paired EOC tissues from omentum or peritoneum metastases and primary EOC tissues was examined by western blot. (B) The protein expression of EGFR in HO-8910 and HO-8910pm cell lines was examined by western blotting. (C) The expression level of miR-7 in HO-8910pm and ES-2 cells transfected with miR-7 or NC was analyzed by qRT-PCR. (D) The expression level of EGFR mRNA in HO-8910pm and ES-2 cells transfected with miR-7 or NC was analyzed by qRT-PCR. (E) The expression of EGFR protein was analyzed by western blotting in HO-8910pm and ES-2 cells transfected with miR-7 or NC, Glyceraldehyde 3-phosphate dehydrogenase (GAPDH) was used as an internal control. (*P<0.05. **P<0.01).

To detect the underlying mechanism by which miR-7 inhibit EOC invasion and metastasis, we searched for miR-7 targets using such as TargetScan and Pictar. That identified 181 candidate target genes including EGFR. It's demonstrated that miR-7 downregulates EGFR expression by directly targeting EGFR mRNA 3′-UTR [17]. We were particularly interested in EGFR because of its positive roles in cancer cell migration and invasion and its overexpression in EOC. We further investigated the expression and significance of EGFR in EOC metastasis by detecting EGFR protein expression in 17-paired metastatic EOC tissues and primary EOC tissues. Western blotting analysis showed that tissues from omentum or peritoneum metastases expressed higher levels of EGFR compared with primary EOC tissues, indicating a positive correlation between the expression of EGFR and the metastatic status of EOC tissues (Figure 2A, Figure S1A). Meanwhile, we also detected EGFR protein expression by western blotting in both HO-8910 and HO-8910pm cells. We found that EGFR expression was significantly increased in highly metastatic clone HO-8910pm compared with HO-8910 (Figure 2B, Figure S1B). Taken together, our results suggest that EGFR expression is positively correlated with EOC metastasis and that EGFR might promote EOC progression.

To make sure the roles of miR-7 in regulation of EGFR expression in EOC cells, both HO-8910pm and ES-2 cells were transfected with miR-7 plasmid or miR-NC. qRT-PCR showed that miR-7 expression was significantly increased (Figure 2C) whereas EGFR mRNA expression was significantly decreased (Figure 2D) after miR-7 transfection in both cell lines. Furthermore, Western blotting analysis indicated that EGFR protein expression was significantly decreased after miR-7 transfection in both HO-8910pm and ES-2 cells (Figure 2E, Figure S1C). Our results suggest that miR-7 downregulates EGFR in EOC.

### miR-7 suppresses EOC cell invasion and migration in vitro

**Figure 3:**
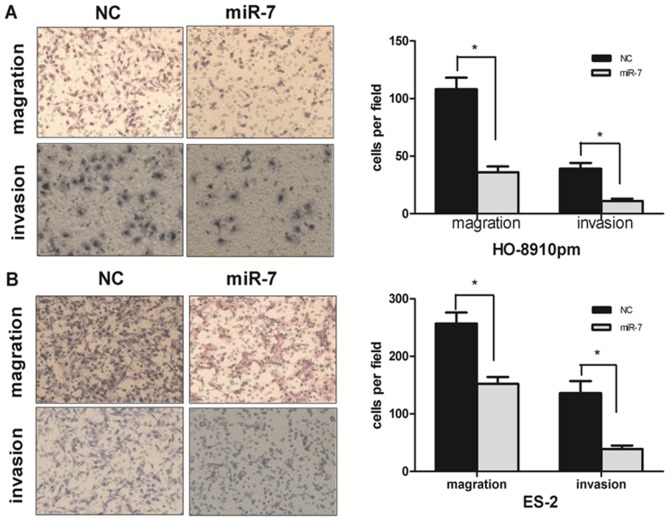
miR-7 suppresses EOC cell invasion and migration in vitro. (A) Transwell migration and invasion assays using HO-8910pm cells transfected with miR-7 or negative control (NC). Representative images are shown on the left, and the quantification of 6 randomly selected fields is shown on the right. (B) Transwell migration and invasion assays using ES-2 cells transfected with miR-7 or NC. Representative images are shown on the left, and the quantification of 6 randomly selected fields is shown on the right. The values shown are expressed as the means ± SD. (*P<0.05.**P<0.01).

To investigate whether miR-7 regulates cell invasion and migration in EOC, both HO-8910pm and ES-2 cells were transfected with miR-7 plasmid or miR-NC. Transwell migration and invasion assays were then performed. We found that transfection with miR-7 significantly suppressed the invasion of both HO-8910pm cells (Figure 3A) and ES-2 cells (Figure 3B). Similarly, migration capacity was also significantly down-regulated in both HO-8910pm-miR-7 cells and ES-2-miR-7 cells versus HO-8910pm-NC cells and ES-2-NC cells (Figure 3A, Figure 3B). These results indicate that miR-7 participates in the regulation of cell migration and invasion in EOC.

### Overexpression of miR-7 reverses EMT in EOC cell

**Figure 4:**
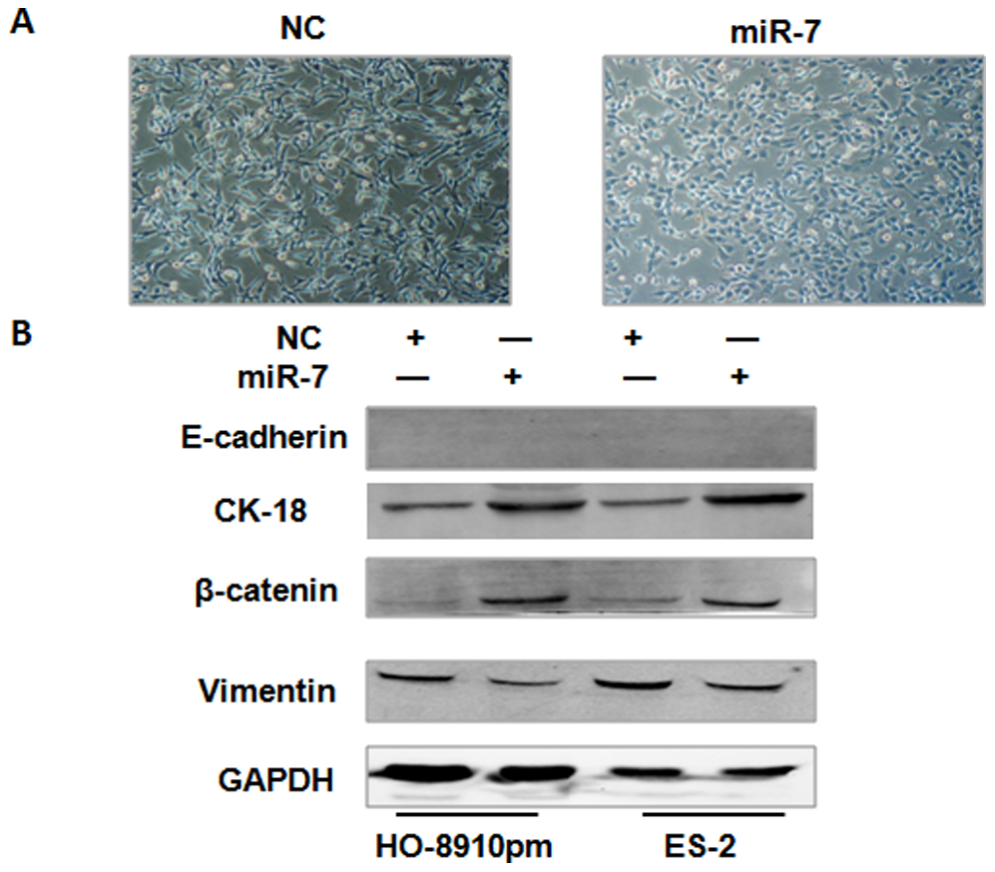
Overexpression of miR-7 reverses EMT in EOC cell. (A) Phase-contrast images of ES-2 cells infected with the miR-7 or NC. (B) The protein expression of E-cadherin, CK-18, β-catenin and Vimentin in HO-8910pm and ES-2 cells transfected with miR-7 or NC were examined by western blotting, GAPDH was used as an internal control. (*P<0.05. **P<0.01).

In EOC cells, we observed that miR-7 transfection resulted in morphological changes from an elongated, spindle-shaped, mesenchymal phenotype to a more rounded, epithelial-like phenotype, with cells aggregating in groups (Figure 4A). These changes represent the reverse processes of EMT, which is an important reason for epithelial cancer to gain the ability of invasion and metastasis. Furthermore, we examined the protein expression of the epithelial markers E-cadherin, CK-18 and β-catenin, as well as the mesenchymal marker vimentin by Western blotting. Although there were no E-cadherin expression in both cell lines even after miR-7 transfection, CK-18 and β-catenin expression dramatically increased, whereas vimentin expression decreased in both HO-8910pm and ES-2 cells after miR-7 transfection (Figure 4B, Figure S2). Our data suggest that overexpression of miR-7 reverses EMT in EOC cell lines.

### Luciferase reporter assays

**Figure 5:**
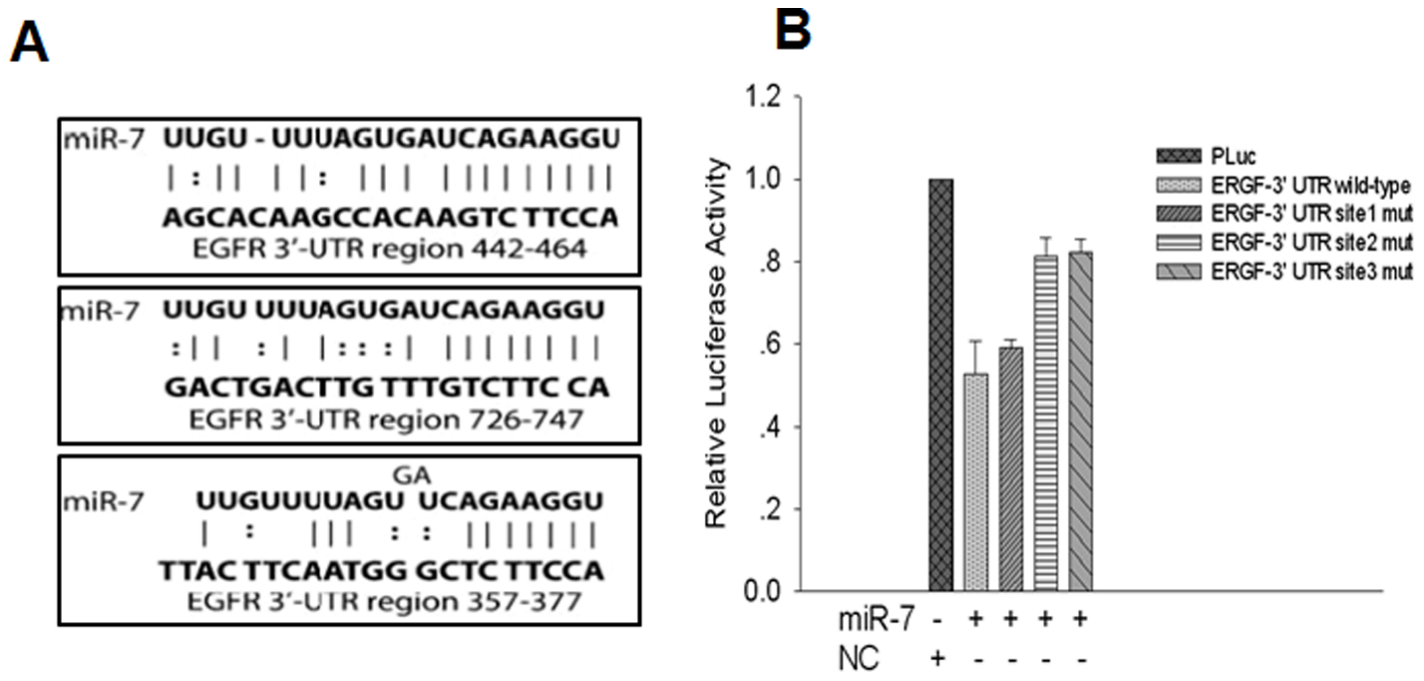
miR-7 downregulate EGFR by interaction with 3′UTR. (A) Diagram of EGFR 3′UTR reporter construct. (B) The wild type or mutant reporter plasmids were cotransfected with miR-7 or NC in ES-2. (*P<0.05. **P<0.01).

To understand the molecular mechanism by which miR-7 suppress EOC invasion and metastasis, we used different computational methods to search for miR-7 targets, such as Targetsan and pictar. These methods found 181 possible candidate genes. We were particularly interested in EGFR because of its positive roles in cancer cell invasion. Analysis of the 3′-UTR sequence of EGFR indentified three possible binding sites for miR-7. To determine whether EGFR is direct target of miR-7, we constructed its 3′-UTR fragments, in which wild-type and mutant binding sites were inserted into the region immediatedly downstream of the reporter gene (Figure 5A), luciferase reporter assays showed that miR-7 transfection caused a remarkable decrease in luciferase activity which contained wild-type 3′-UTR fragments binding sites (Figure 5B).

### miR-7 suppresses AKT and ERK1/2 pathway activation dependent of its EGFR inhibition in EOC cells

**Figure 6:**
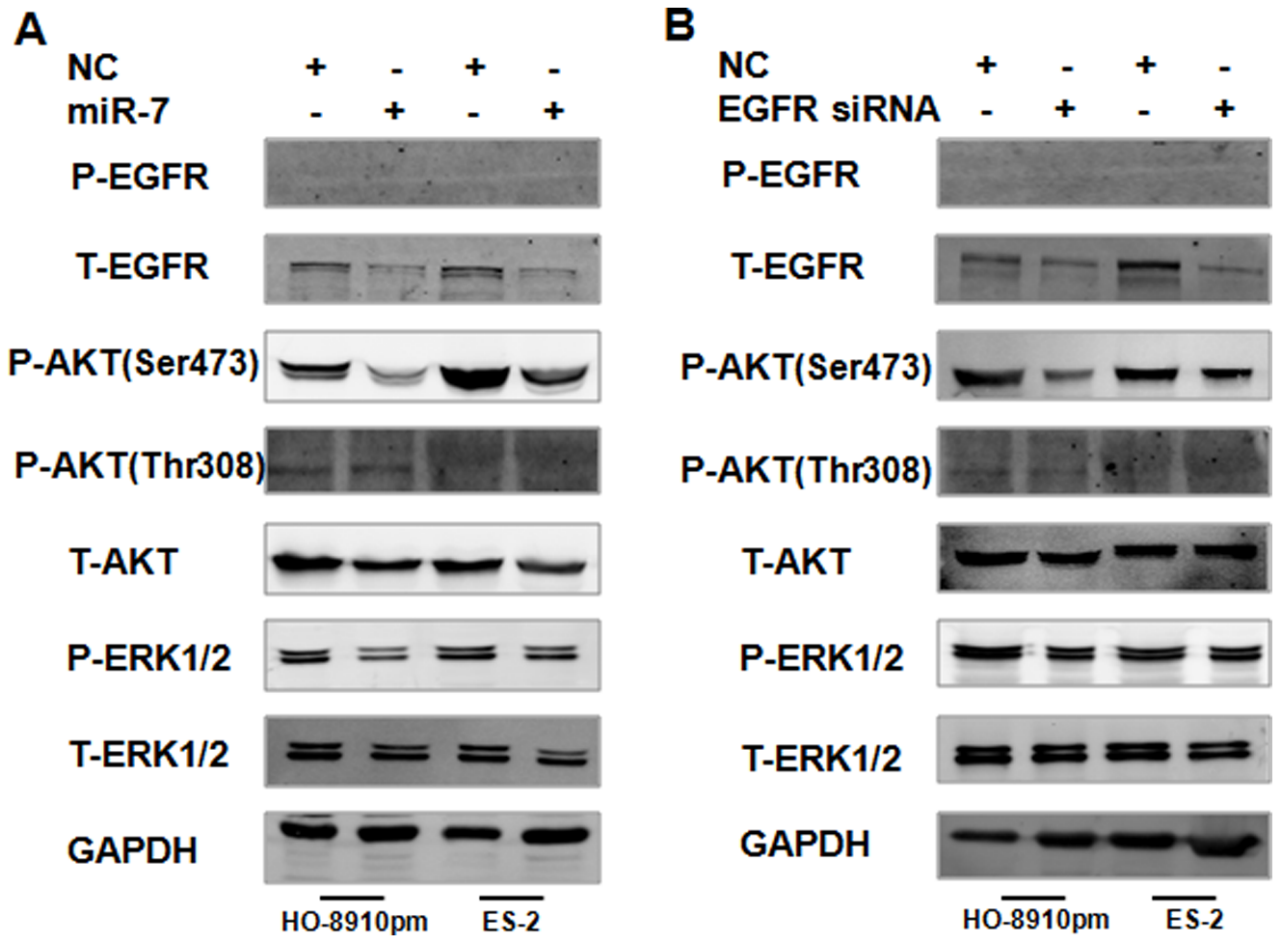
miR-7 suppresses AKT and ERK1/2 pathway activation dependent of its EGFR inhibition in EOC cells. (A) HO-8910pm and ES-2 cells were transfected with miR-7 or NC, EGFR, AKT and ERK1/2 phosphorylation were analyzed by western blotting. (B) HO-8910pm and ES-2 cells were transfected with EGFR siRNA or NC, EGFR, AKT and ERK1/2 phosphorylation were analyzed by western blotting. (*P<0.05. **P<0.01).

Previous studies showed that EGFR regulates AKT and ERK1/2 activity in ovarian cancer [15], [22]. Because miR-7 can post-transcriptionally inhibit the expression of EGFR, we hypothesized that miR-7 regulates AKT and ERK1/2 pathway activation. To investigate the effect of miR-7 on AKT and ERK1/2, we transfected HO-8910pm and ES-2 cells with miR-7 plasmid or miR-NC, and then examined phosphorylation of AKT and ERK1/2 by western blotting. Transfection with miR-7 suppressed phosphorylation of AKT at Ser473 and ERK1/2 at Thr202/Tyr204, however, miR-7 did not significantly alter AKT phosphorylation at Thr308 (Figure 6A). Since the activation of EGFR is playing major role in the metastasis of many cancers, we also detected its phosphorylation status after miR-7 transfection in HO-8910pm and ES-2 cells. However, in our studies, there was no EGFR phosphorylation pre and post miR-7 transfection, whereas miR-7 transfection did inhibit total EGFR protein expression (Figure 6A, Figure S3 (A-C)). As evidence that miR-7 has many target genes, we sought to determine whether miR-7 mediated AKT and ERK1/2 pathway activation is dependent of its EGFR inhibition. HO-8910pm and ES-2 cells were transfected with EGFR siRNA or the negative control. We showed that silencing of EGFR with this siRNA in ovarian cancer cells decreased phosphorylation of AKT at Ser473 and ERK1/2 at Thr202/Tyr204 on Western Blotting, but there was no significant change in phosphorylation of AKT at Thr308 (Figure 6B, Figure S3 (D-E)).

### miR-7 reverses EMT through EGFR/AKT and EGFR/ERK1/2 pathway

**Figure 7:**
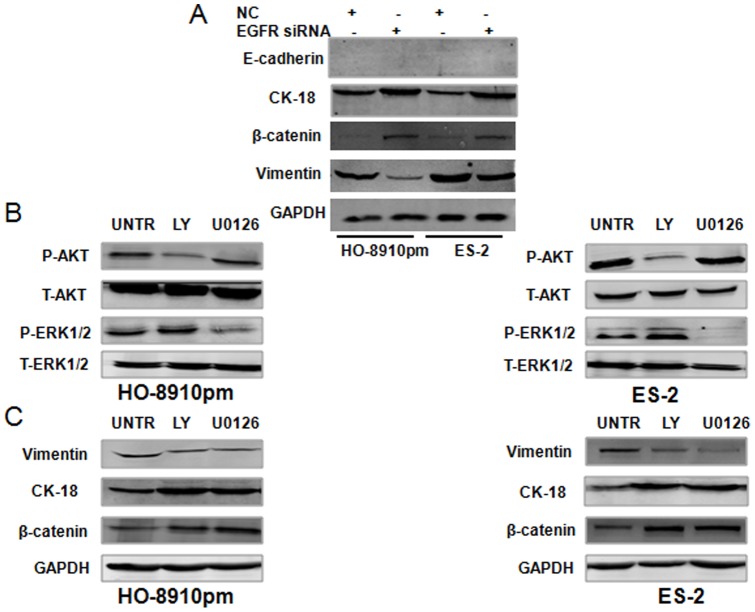
miR-7 reverses EMT through EGFR/AKT and EGFR/ERK1/2 pathway. (A) HO-8910pm and ES-2 cells were transfected with EGFR siRNA or NC, the protein expression of E-cadherin, CK-18, β-catenin and vimentin were explored by western blotting. (B) HO-8910pm and ES-2 cells were treated with LY294002 (20 umol/l) or U0126 (10 umol/l), AKT and ERK1/2 phosphorylation were analyzed by western blotting. (C) HO-8910pm and ES-2 cells were treated with LY294002 (20 umol/l) or U0126 (10 umol/l), the protein expression of CK-18, β-catenin and vimentin were explored by western blotting. GAPDH was used as an internal control (*P<0.05. **P<0.01).

To determine whether the EGFR/ AKT and EGFR/ERK1/2 signaling pathways were involved in the reversal of EMT by miR-7, we transfected EGFR siRNA into HO-8910pm and ES-2 cells and then explore the protein expression of E-cadherin, CK-18, β-catenin and vimentin by western blotting. We found that there was no E-cadherin expression pre and post EGFR siRNA transfection in both cell lines, however, CK-18 and β-catenin expression dramatically increased, whereas vimentin expression decreased in both HO-8910pm and ES-2 cells after EGFR siRNA transfection (Figure 7A, Figure S4 (A-C)). Then we used the PI3K/AKT inhibitor LY294002 and the ERK1/2 inhibitor U0126 to specifically block the Akt and the ERK1/2 pathways, respectively. As expected, the PI3K/AKT inhibitor LY294002 inhibited AKT phosphorylation, while ERK1/2 inhibitor U 0126 inhibited ERK1/2 phosphorylation in HO-8910pm and ES-2 cells (Figure 7B, FigureS4 (D-G)). Further, both the AKT and ERK1/2 inhibitors enhanced CK-18 and β-catenin expression and suppressed vimentin expression in HO-8910pm and ES-2 cells (Figure 7C and Figure S4 (H-M)). Our data demonstrated the involvement of the EGFR/ AKT and EGFR/ERK1/2 pathways in the miR-7 reversed EMT in ovarian cancer cells.

### miR-7 and EGFR are inversely expressed in EOC primary and metastasis tissues

**Figure 8:**
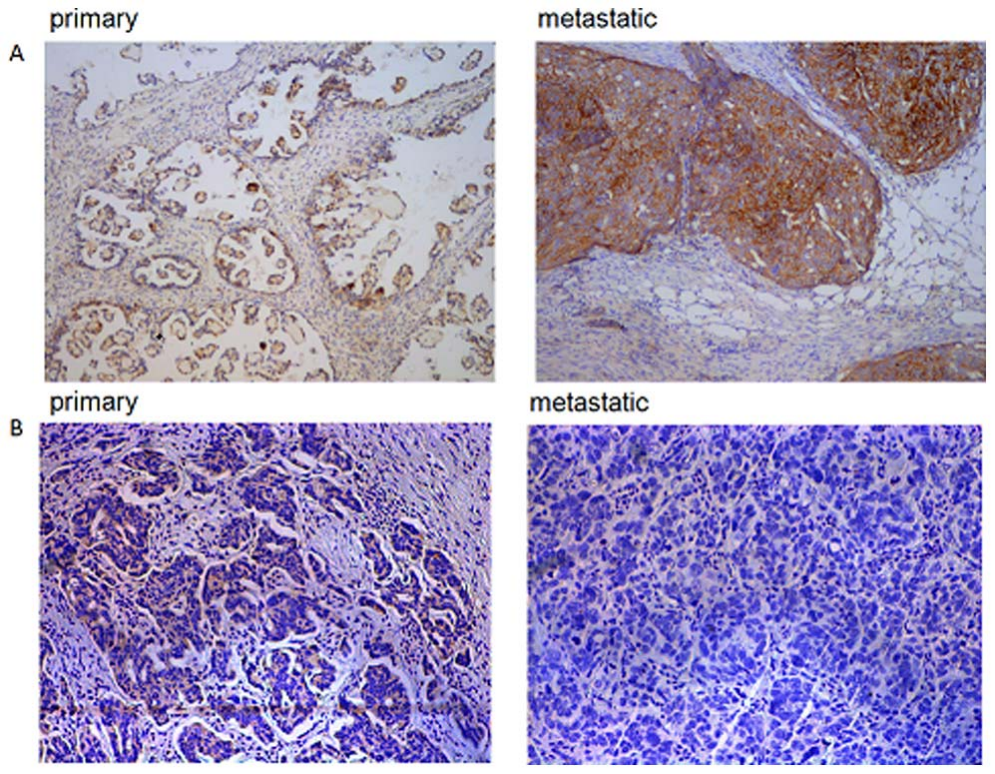
miR-7 and EGFR are inersely expressed in EOC tissues. (A) Expression of miR-7 in primary EOC and its matched metastatic tissue by CISH. (B) Expression of EGFR in primary EOC and its matched metastatic tissue by IHC. (*P<0.05. **P<0.01).

We used CISH and IHC to detect the expression of miR-7 and EGFR in the same type of tissue. We determined whether miR-7 expression was associated with EGFR expression in EOC tissuses. The tissue contained 25 pairs of primary EOC tissues and their matched metastatic tissues. The CISH analysis showed an overt reduction of miR-7 in the metastatic tissues compared with their corresponding primary EOC tissues (Figure 8A, Table 1). By contrast, IHC results revealed that EGFR expression was higher in metastatic tissues than in primary EOC tissues (Figure 8B, Table 2). Furthermore, statistical analysis showed that EGFR expression was inversely correlated with miR-7 expression (Table 3).

**Table 1:** miR-7 expression in EOC tissues and matched metastases.

	miR-7 expression level				
	(–)	(+)	(++)	(+++)	P-values
EOC	2/25	6/25	12/25	5/25	-
Metastasis	14/25	6/25	0/25	0/25	<0.05

H-score =  proportion score×intensity score. A total score of 0–12 was calculated and graded as negative (−, score:0), weak (+, score:1–4), moderate (++, score:5–8) or strong (+++, score:9–12).

**Table 2:** EGFR expression in EOC tissues and matched metastases.

	EGFR expression level				
	(−)	(+)	(++)	(+++)	P-values
EOC	4/25	15/25	5/25	1/25	-
metastasis	2/25	9/25	10/25	4/25	<0.05

H-score =  proportion score×intensity score. A total score of 0–12 was calculated and graded as negative (−, score:0), weak (+, score:1–4), moderate (++, score:5–8) or strong (+++, score:9–12).

**Table 3:** Correlation between the expression of EGFR protein and miR-7 in 25 pairs of EOC and their matched metastases.

EGFR	miR-7				n	P-values	Spearman's	
	(−)	(+)	(++)	(+++)			P	R
(−)	1	5	0	0	50	<0.05	<0.01	−0.3199
(+)	8	1	11	4	_	_	_	_
(++)	7	4	1	1	_	_	_	_
(+++)	5	2	0	0	_	_	_	_

H-score =  proportion score×intensity score. A total score of 0–12 was calculated and graded as negative (−, score:0), weak (+, score:1–4), moderate (++, score:5–8) or strong (+++, score:9–12).

## Discussion

miRNAs have emerged as critical regulators of carcinogenesis and malignant progression of cancer by targeting oncogenes and tumor suppressor genes [23], [24]. It has been implicated that miR-7 inhibits tumor invasion and metastasis in glioblastoma, schwannoma, lung cancer, breast cancer and many other cancers [25], [26], [27]. In the present study, we investigated the biological role of miR-7 in human EOC metastasis. We found that miR-7 was down-regulated in metastatic epithelial ovarian cancer (EOC) tissues compared with primary EOC tissues. miR-7 expression was also significantly decreased in highly metastatic clone HO-8910pm compared with HO-8910. Furthermore, we showed that the expression of miR-7 was lowest in ES-2 cell line, which is another EOC cell with highly metastatic potential among seven EOC cell lines (HO-8910pm, HO-8910, A2780, A2780/DDP, SKOV-3, CAOV-3 and ES-2). Therefore, our findings indicated that miR-7 expression is inversely correlated with EOC metastasis.

Interestingly, each miRNA can potentially regulate hundreds of genes at post-transcriptional levels by binding to the specific sequences in the target mRNA molecules based on ‘imperfect complementarity’. It has been shown that overexpression of miR-7 inhibited tumor invasion and metastasis by targeting insulin-like growth factor-1 receptor (IGF-1R) in gastric cancer [9]. miR-7 has also been reported to target FAK in breast cancer cells [28]. In recent years, mounting evidence indicates that EGFR is a direct and functional target of miR-7 [10], [17]. Receptor tyrosine kinase of the EGFR family regulates essential cellular functions, including proliferation, survival, migration, and differentiation. Published data demonstrates that EGFR plays an important role in tumor progression [12], [27]. In this study, we found that tissues from omentum or peritoneum metastases expressed higher levels of EGFR compared with primary EOC tissues. Meanwhile, we found that EGFR was upregulated in highly metastatic clone HO-8910pm compared with HO-8910. These results suggest that EGFR is closely related to ovarian cancer metastasis. Our previous studies also showed that EGFR overexpression and activation result in increased metastasis of human ovarian cancer cells [18], [29]. However, no study is performed to determine whether miR-7 targets EGFR to modulate the behavior of EOC metastasis. Here, we showed that both EGFR mRNA and EGFR protein expression was significantly decreased after miR-7 transfection in EOC cell lines. We subsequently identified that both invasion and migration capacity were significantly down-regulated after miR-7 transfection in EOC in vitro. Our present experimental results confirmed for the first time that miR-7 suppressed EOC cell invasion and migration. Taken together, our results indicate that miR-7 suppresses cell invasion and migration at least in part, through direct targeting of EGFR in EOC.

EMT refers to a biological process that the epithelial cells transform into mesenchymal cells through the specific program, which plays an important role in embryonic development, chronic inflammation, organize rebuilding, cancer metastasis and a variety of fibrosis disease [30], [31], [32]. Epithelial cells undergoing EMT towards a more mesenchymal phenotype involves decreased expression of epithelial markers ( E-cadherin, β-catenin, CK-18) and increased expression of mesenchymal markers (N-cadherin, vimentin) [31], [33], [34]. They become more invasive by expressing proteases that allow them to pass through the underlying basement membrane and migrate, both being critical steps in the multi-step process of metastasis. It has been reported that some miRNAs such as miR-200, miR-429 and miR-141 can promote or suppress cancer metastasis and invasion by regulating EMT [35], [36]. Recent study showed that miR-155 is upregulated during TGF-β-induced EMT [37], [38]. In the present study, we observed that overexpression of miR-7 induced morphological changes from an elongated, spindle-shaped, mesenchymal phenotype to a more rounded, epithelial-like phenotype, with cells aggregating in groups in EOC cells. Although there were no E-cadherin expression in both HO-8910pm and ES-2 cell lines even after miR-7 transfection, epithelial markers CK-18 and β-catenin expression were dramatically increased but mesenchymal marker vimentin expression was decreased after miR-7 transfection. Our data suggest that miR-7 plays important roles in reversing EMT and inhibiting EOC metastasis.

Our work with EGFR pathway in ovarian cancer [18], [29], [39] led us to investigate whether the EGFR and its downstream signaling pathways were involved in the reversal of EMT by miR-7. The EGFR exerts its function in the cellular environment mainly, if not exclusively, via its tyrosine. Existing data indicated that the effects of EGFR signaling on cell migration and invasion are mediated by PI3K/AKT and ERK1/2 signaling pathways in EOC [15], [40], [41], [42]. It has been shown that antagonism of miR-21 reversed EMT, accompanied with PTEN up-regulation and AKT/ERK1/2 inactivation. The inhibitors of PI3K-AKT and ERK1/2 pathways, LY294002 and U0126 significantly suppressed EMT, indicating that AKT and ERK1/2 pathways are required for miR-21 mediating EMT in breast cancer [27]. In the present study, we have demonstrated that miR-7 suppressed both AKT and ERK1/2 phosphorylation in EOC cells. Meanwhile, overexpression of miR-7 resulted in the reduced expression of total EGFR protein without any change in the phosphorylation state of EGFR. Therefore, we sought to further show the effects of miR-7 on both Akt and ERK1/2 activation were via EGFR inhibition. To address this, we determined the effects on both Akt and ERK1/2 phosphorylation of transfecting the same ovarian cancer cell lines with an EGFR siRNA. Similar to miR-7 transfection, silencing of EGFR with this siRNA blocked phosphorylation of both Akt and ERK1/2, which support the contention that miR-7 decreased both Akt and ERK1/2 activation dependent of its effects on EGFR inhibition. Our results indicate that miR-7 exerted its effects in human ovarian cancer cells via the inactivation of the EGFR/AKT and EGFR/ERK1/2 signaling pathway. The activation of PI3K/AKT signaling has been found to stimulate Snail and Slug expression via GSK-3b/β-catenin signaling and to subsequently promote EMT in different cellular contexts [43], [44]. Recent study showed that Gab2 overexpression, via activation of the PI3K-ZEB1 pathway, promotes characteristics of EMT in ovarian cancer cells [45]. More recently it has been demonstrated that EGF induces serous borderline ovarian tumors cell migration and invasion by activating EMT, which involves the activation of the ERK1/2 and PI3K/Akt pathways and, subsequently, Snail, Slug and ZEB1 expression [22]. Here, we observed that silencing of EGFR increased CK-18 and β-cadherin expression and decreased vimentin expression, which confirmed that EGFR is a target of miR-7 in reversing EMT. Furthermore, inhibition of both AKT and ERK1/2 signaling results in upregulation of CK-18 and β-catenin expression and downregulation of vimentin expression in EOC cells. Taken together, our data first demonstrated the involvement of the EGFR/ AKT and EGFR/ERK1/2 pathways in the miR-7 reversed EMT in ovarian cancer cells.

In summary, our results demonstrated that miR-7 expression was inversely correlated with EOC metastasis. Further, miR-7 reversed EMT through AKT and ERK1/2 pathway inactivation by reducing EGFR expression in HO-8910pm and ES-2 cell lines. Thus, miR-7 might be a potential prognostic marker and therapeutic target for ovarian cancer metastasis intervention.

## Supporting Information

Figure S1 **The quantitative analysis of Western blot images in** **fig.2** **.** (A) The protein expression of EGFR in 17-paired EOC tissues from omentum or peritoneum metastases and primary EOC tissues was examined by western blot. (B) The protein expression of EGFR in HO-8910 and HO-8910pm cell lines was examined by western blotting. (C) The expression of EGFR protein was analyzed by western blotting in HO-8910pm and ES-2 cells transfected with miR-7 or NC.
(TIF)

Figure S2 **The quantitative analysis of Western blot images in** **fig.4** **.** (A) The protein expression of CK-18 in HO-8910pm and ES-2 cells transfected with miR-7 or NC. (B) The protein expression of β-catenin in HO-8910pm and ES-2 cells transfected with miR-7 or NC. (C) The protein expression of Vimentin in HO-8910pm and ES-2 cells transfected with miR-7 or NC.
(TIF)

Figure S3 **The quantitative analysis of Western blot images in** **fig.6** **.** HO-8910pm and ES-2 cells were transfected with EGFRsiRNA or miR-7. (A/D) T-EGFR were analyzed by western blotting. (B/E) P-AKT were analyzed by western blotting. (C/F) P- ERK1/2 were analyzed by western blotting. (*P<0.05. **P<0.01).
(TIF)

Figure S4 **The quantitative analysis of Western blot images in** **fig.7** **.** HO-8910pm and ES-2 cells were transfected with EGFR siRNA or NC, the protein expression of CK-18(A), β-catenin (B) and vimentin (C) were explored by western blotting. HO-8910pm and ES-2 cells were treated with LY294002 (20 umol/l) or U0126 (10 umol/l), AKT (D/F) and ERK1/2 (E/G) phosphorylation were analyzed by western blotting. HO-8910pm and ES-2 cells were treated with LY294002 (20 umol/l) or U0126 (10 umol/l), the protein expression of vimentin (H/K), CK-18 (I/L) and β-catenin (J/M) were explored by western blotting. GAPDH was used as an internal control(*P<0.05. **P<0.01).
(TIF)
