# Supplementary figures and images for: MicroRNA-7 Inhibits Tumor Metastasis and Reverses Epithelial-Mesenchymal Transition through AKT/ERK1/2 Inactivation by Targeting EGFR in Epithelial Ovarian Cancer
Source: PLoS One. 2014 May 9;9(5):e96718. doi: 10.1371/journal.pone.0096718 (PMC4016102; doi:10.1371/journal.pone.0096718)

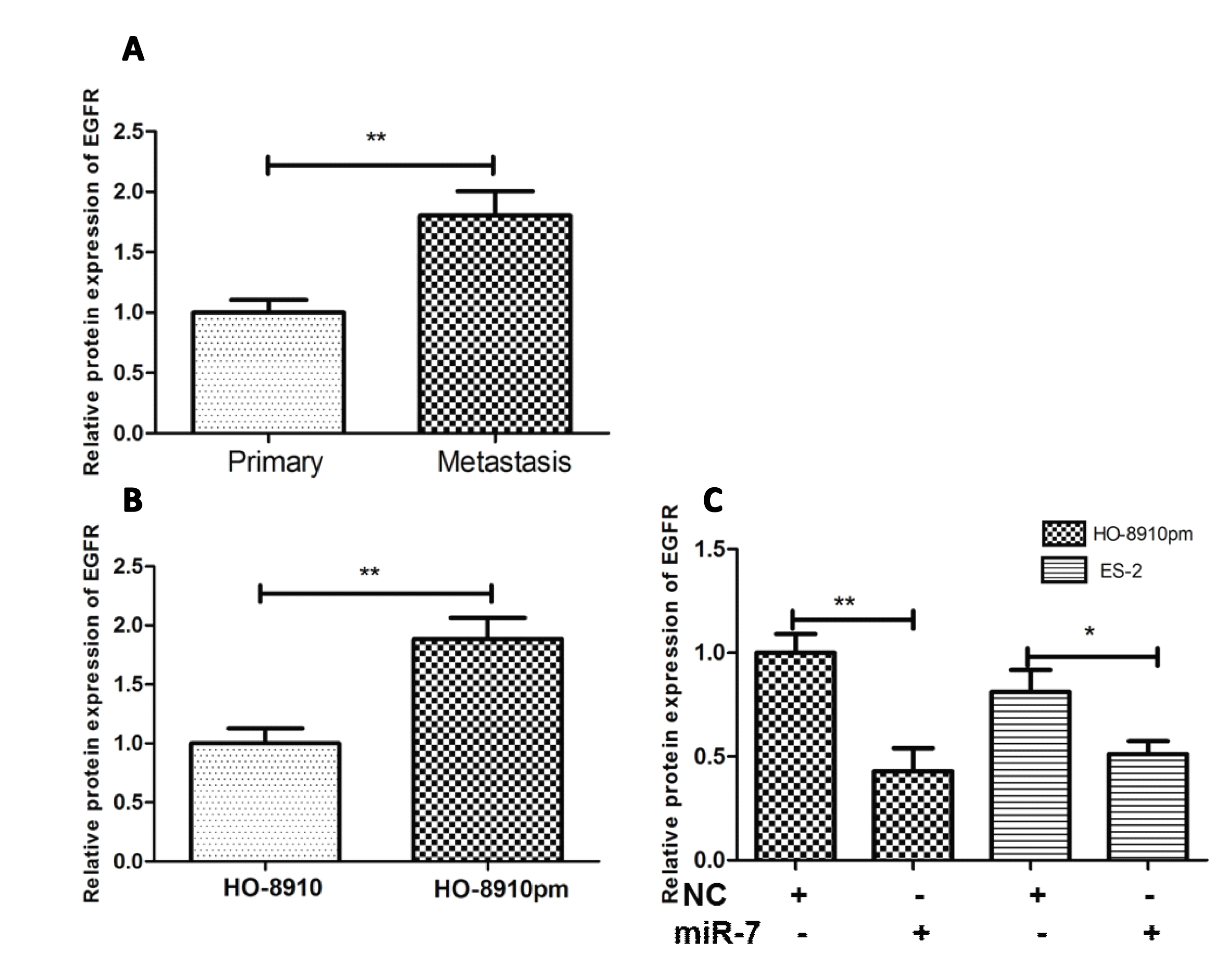

Supplement: Figure S1 — The quantitative analysis of Western blot images in fig.2 . (A) The protein expression of EGFR in 17-paired EOC tissues from omentum or peritoneum metastases and primary EOC tissues was examined by western blot. (B) The protein expression of EGFR in HO-8910 and HO-8910pm cell lines was examined by western blotting. (C) The expression of EGFR protein was analyzed by western blotting in HO-8910pm and ES-2 cells transfected with miR-7 or NC. (TIF) [file pone.0096718.s001.tif]

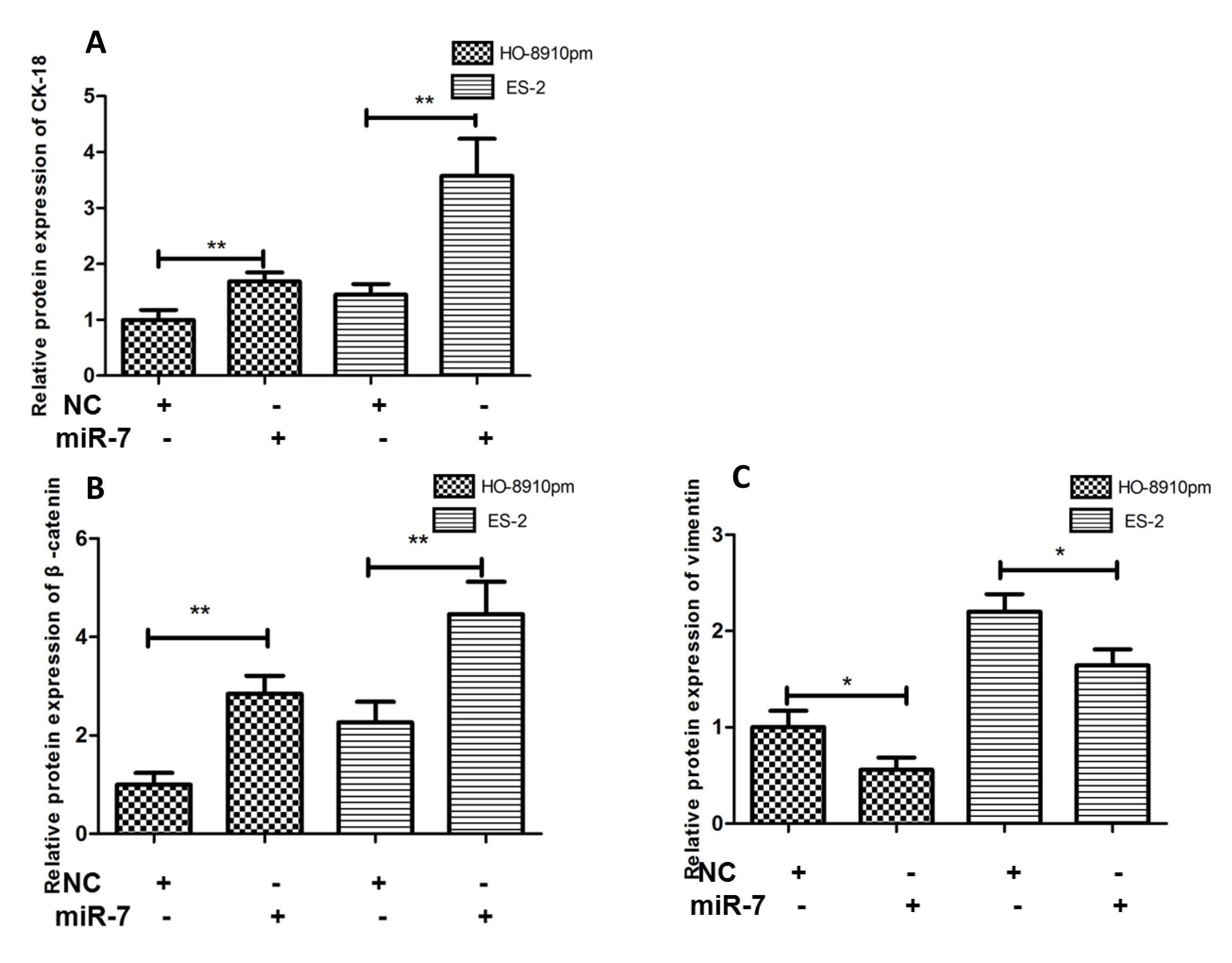

Supplement: Figure S2 — The quantitative analysis of Western blot images in fig.4 . (A) The protein expression of CK-18 in HO-8910pm and ES-2 cells transfected with miR-7 or NC. (B) The protein expression of β-catenin in HO-8910pm and ES-2 cells transfected with miR-7 or NC. (C) The protein expression of Vimentin in HO-8910pm and ES-2 cells transfected with miR-7 or NC. (TIF) [file pone.0096718.s002.tif]

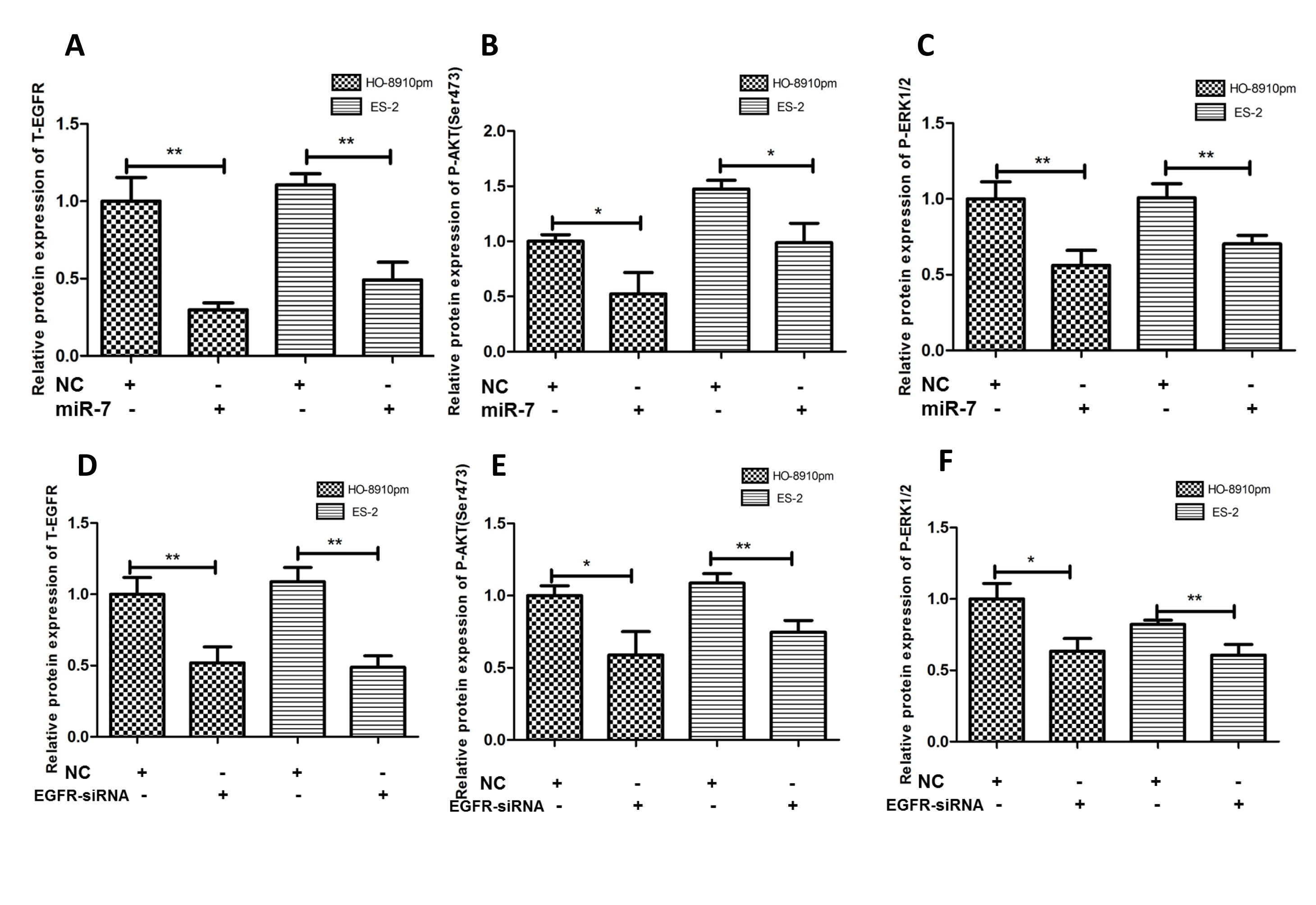

Supplement: Figure S3 — The quantitative analysis of Western blot images in fig.6 . HO-8910pm and ES-2 cells were transfected with EGFRsiRNA or miR-7. (A/D) T-EGFR were analyzed by western blotting. (B/E) P-AKT were analyzed by western blotting. (C/F) P- ERK1/2 were analyzed by western blotting. (*P<0.05. **P<0.01). (TIF) [file pone.0096718.s003.tif]

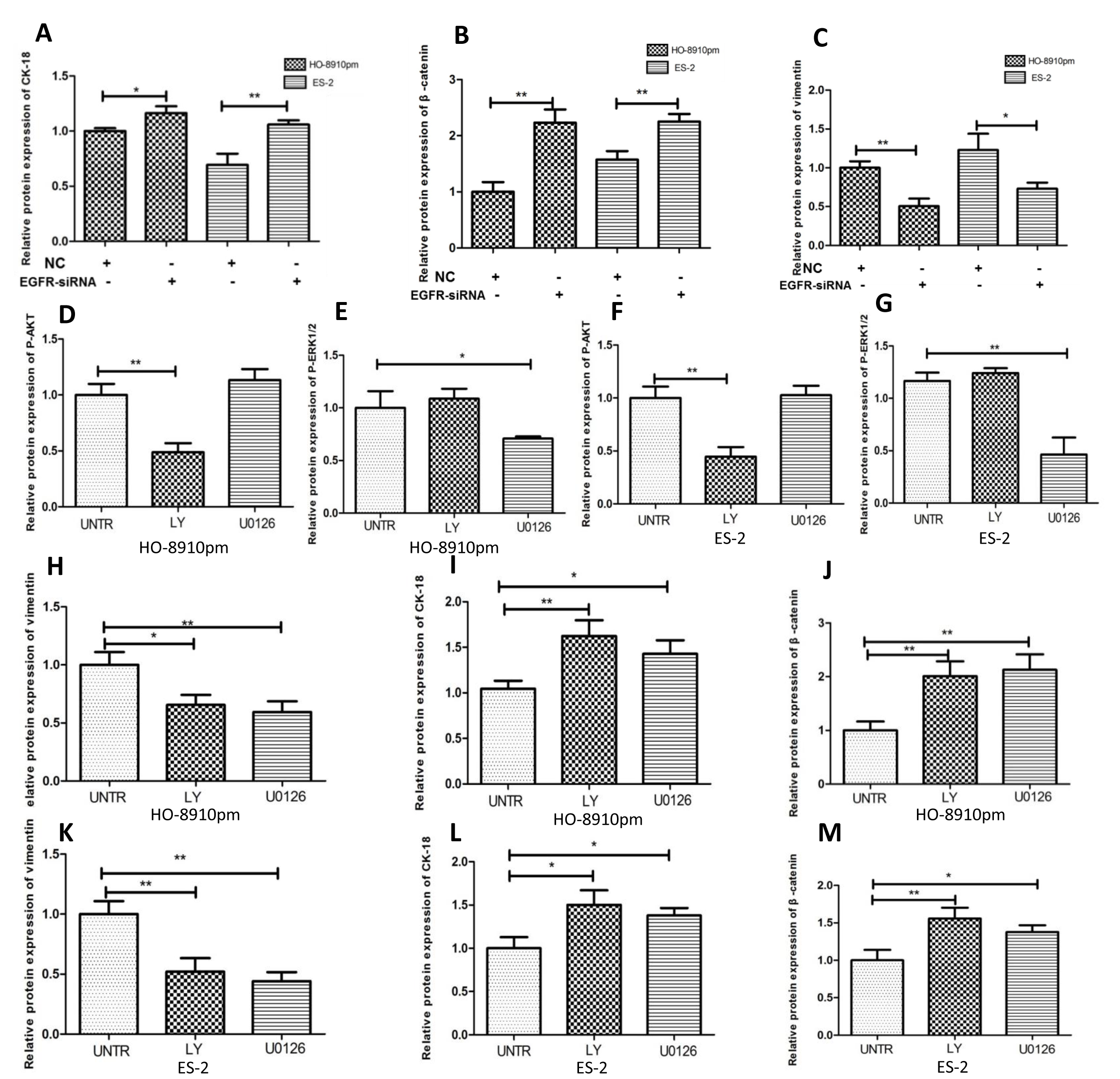

Supplement: Figure S4 — The quantitative analysis of Western blot images in fig.7 . HO-8910pm and ES-2 cells were transfected with EGFR siRNA or NC, the protein expression of CK-18(A), β-catenin (B) and vimentin (C) were explored by western blotting. HO-8910pm and ES-2 cells were treated with LY294002 (20 umol/l) or U0126 (10 umol/l), AKT (D/F) and ERK1/2 (E/G) phosphorylation were analyzed by western blotting. HO-8910pm and ES-2 cells were treated with LY294002 (20 umol/l) or U0126 (10 umol/l), the protein expression of vimentin (H/K), CK-18 (I/L) and β-catenin (J/M) were explored by western blotting. GAPDH was used as an internal control(*P<0.05. **P<0.01). (TIF) [file pone.0096718.s004.tif]
